# Myofunctional orofacial examination tests: a literature review

**DOI:** 10.1186/s12903-023-03056-1

**Published:** 2023-06-02

**Authors:** Delal Dara Kilinc, Duygu Mansiz

**Affiliations:** 1 Independent Researcher, Istanbul, Turkey; 2 Department of Orthodontics, Faculty of Dentistry, Istanbul Aydin University, Istanbul, Turkey

**Keywords:** Stomatognathic system, Myofunctional orofacial evaluation, Orofacial myofunctional examination, Orofacial myofunctional examination test

## Abstract

**Introduction:**

Myofunctional orofacial examination (MOE) is an important tool for the assessment of the stomatognathic system and orofacial functions, and the early diagnosis of orofacial myofunctional disorders. Therefore, the purpose of the study is to scan the literature and determine the most preferred test for myofunctional orofacial examination.

**Materials and Methods:**

A literature review was conducted to collect information. Pubmed and ScienceDirect database was explored by using keywords gained by MeSH (Medical Subject Headings).

**Results:**

Fifty-six studies were retrived from the search and all of the studies were screened and evaluated regarding the subject, aim, conclusions and the orofacial myofunctional examination test they used. It has been observed that traditional evaluation and inspection methods have been replaced by newer and methodological approaches in recent years.

**Conclusions:**

Although the few tests used differ, 'Orofacial Examination Test With Scores’ (OMES) was found to be the most preferred myofunctional orofacial evaluation method from ENT to cardiology.

## Introduction

Myofunctional orofacial anomalies refer to abnormal orofacial functions that can lead to changes in the function, structure and formation of the stomatognathic system. These disorders can cause malocclusions of the jaws, temporomandibular joint disorders, and other problems involving the orofacial region. Factors triggrering malocclusions may include myofunctional anomalies characterized by disturbances in chewing, swallowing and breathing patterns. Infantile swallowing, mouth breathing and tongue thrust, which are clinically common, and can be caused by genetic and/or environmental factors could be counted as examples [1–3]. The prevalence of myofunctional orofacial anomalies in the general population is presented as 38% [4].

Orthodontic and dentofacial orthopedic treatment is often used to correct malocclusions and other orofacial abnormalities [5–7]. But the relationship between myofunctional anomalies and orthodontics is complex and still not exact clear. Several studies have investigated the relationship between myofunctional anomalies and orthodontics, and found that orthodontic treatment can improve orofacial function in patients with myofunctional anomalies [8–12]. However, other studies have suggested that orthodontic treatment can have negative effects on stomatognathic functions in some patients [13, 14].

The diagnosis of myofunctional orofacial disorders requires a comprehensive myofunctional orofacial examination (MOE) of the orofacial musculature and function. This examination is commonly performed by speech-language pathologists, dentists, and orthodontists. The MOE has been described in several studies as a comprehensive tool for assessing the orofacial region by various examination protocols. However, there is no global consensus on a standardized orofacial myofucntional examination form. Various examination forms and methods have been presented for the evaluation of orofacial anomalies and functions which includes the examination of stomagnatic region: orofacial muscles, lips, tongue, breathing, swallowing patterns, cranio-oro-facial posture and speech. The purpose of the myofunctional orofacial examination is to evaluate the function and structure of the orofacial complex statically and dynamically and, to detect any dysfunction that may be present. The information obtained from the examination is aimed to be used for developing an individualized treatment plan to address any orofacial dysfunction that is detected [15–22].

Myofunctional examination may include the following stages: reviewing the patient's both current health and medical/dental history; statically and dynamically examining and both photographing and video recording of facial/mouth structures, oral functions, and general face and body posture; things to be evaluated sequentially and individually: Respiration and Respiratory tract; Oral Habits; Craniofacial/orofacial appearance (ie, symmetry, posture, growth patterns) and function of involved muscles and TMJ; Evaluation of tongue (posture, structure, function, tongue ties); hard and soft tissues of the mouth; occlusion; speaking function, appearance and resting position; chewing; liquid and solid swallowing; review of examination findings; consulting if necessary; establishing a treatment strategy and plan [15–22]. In recent years, the importance of holistic examination and detailed examination has begun to be understood [23].

In addition to the examination, studies are also carried out on the treatment of these anomalies. Rehabilitation of the tongue [24], examination of the TMJ and its effects on occlusion and the orofacial component [25], evaluation of the orofacial muscles and related body muscles to maintain regular functioning [26], and publications presenting the evaluation of chewing and masticatory muscles [27] are some examples.

Nevertheless, the need for a standard and valid myofunctional orofacial assessment test has become more evident for dentists, all specialist dentists and especially orthodontists. That is why, the research question of this study is 'Which is the most preferred and presented myofascial orofacial examination test in the literature?' And thus, the aim of the study is to scan the literature with this point of view and to identify the most valid and used test.

## Methodology

### Information sources and search

Pubmed and ScienceDirect database was searched by one researcher of the present study on April 2, 2023 by using keywords: “myofunctional[Title] AND orofacial[Title] AND examination[Title] AND orofacial[Title] AND myofunctional[Title] AND examination[Title]”, “myofunctional[Title] AND orofacial[Title] AND assessment[Title] AND orofacial[Title] AND myofunctional[Title] AND assessment[Title]”, “(myofunctional AND orofacial AND evaluation) OR (orofacial AND myofunctional AND evaluation)” for PubMed and, "myofunctional orofacial assessment”,”myofunctional orofacial evaluation”, "myofunctional orofacial examination”,”orofacial myofunctional examination”,”orofacial myofunctional assessment”,”orofacial myofunctional evaluation” for ScienceDirect. Medical Subject Headings (MeSH) terms was used in creating the search keywords.

### Study selection

**Fig. 1:**
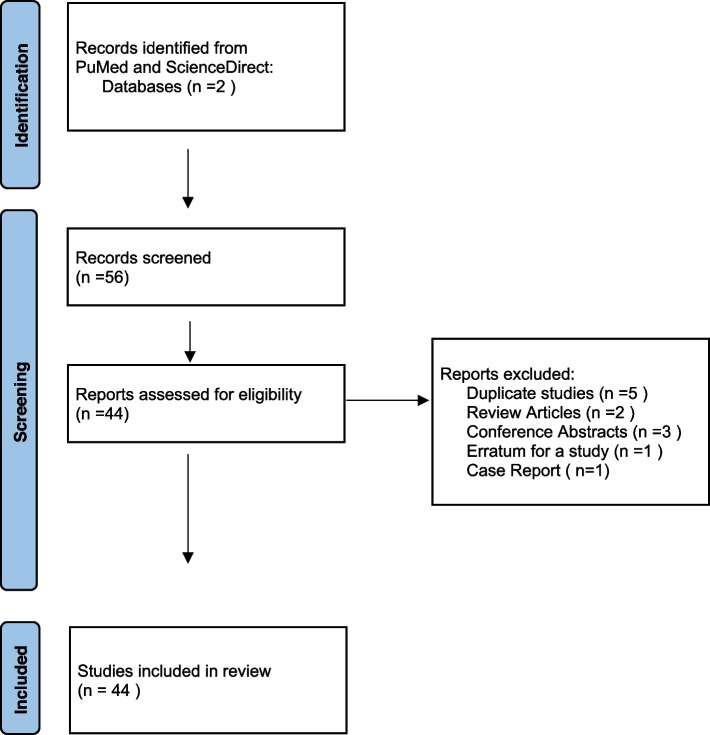
Flow chart of the study

Fifty-six studies reached by screening both databases. 5 studies were duplicates, 2 were review articles, 3 were conference abstracts, 1 was an erratum, 1 was a case report. After excluding those 12 studies, 44 studies remained for evaluation. All of 44 remaining studies were included in screening (Fig. 1). Scanning and data collection was done by an orthodontist (DDK) with 20 years of experience.

### Data extraction and data items

The following data were recorded: database, name of publication, study type, name of the examination form used in the study. In addition all studies were screened regarding subject, aim, conclusions in the discussion of the present study (Table 1).

**Table 1:** Data about the studies screened in the study

**PubMed**		**PubMed**		**PubMed**		**ScienceDirect**		**ScienceDirect**		**ScienceDirect**		**ScienceDirect**		**ScienceDirect**		**ScienceDirect**	
**myofunctional[Title] AND orofacial[Title] AND assessment[Title] OR orofacial[Title] AND myofunctional[Title] AND assessment[Title]**		**myofunctional[Title] AND orofacial[Title] AND examination[Title] OR orofacial[Title] AND myofunctional[Title] AND examination[Title]**		**(myofunctional AND orofacial AND evaluation) OR (orofacial AND myofunctional AND evaluation)**		**"myofunctional orofacial assessment"**		**"myofunctional orofacial evaluation”**		**"myofunctional orofacial examination”**		**"orofacial myofunctional assessment"**		**"orofacial myofunctional evaluation”**		**"orofacial myofunctional examination”**	
**Orofacial myofunctional assessment and quality of life of individuals with Parkinson's disease**	*Research Article—One examination form used in the study: Orofacial Myofunctional Evaluation with Scores (OMES) for Elders*	**ERRATUM: MMBGR Protocol—infants and preschoolers: myofunctional orofacial clinic examination**	*Erratum for MMBRG Protocol for Infants and Preschoolers*	**No items found**	-	**Neuromodulation: A combined-therapy protocol for speech rehabilitation in a child with cerebral palsy**	Research Article, Speech tests, Cognitive tests and Orofacial Myofunctional Evaluation with Scores (OMES) were used in the study	**An index for the evaluation of 3D masticatory cycles stability**	*Research Article Masticatory Stability Index, (MSI) and Orofacial myofunctional assessment protocols (OMES) were used*	**No results found**	-	**Congenital heart disease in children: Orofacial myofunctional aspects, eating behavior and facial temperature**	*Research Article A questionnaire to assess eating behaviors (Montreal Children's Hospital Feeding Scale) and Expanded orofacial myofunctional assessment protocol (OMES-E) and Child Language Test (ABFW), and Thermography infrared of facial temperature protocols were used*	**Protocol of orofacial myofunctional evaluation with scores**	*Research Article Traditional orofacial myofunctional evaluation (TOME), and Orofacial myofunctional evaluation with scores (OMES) were used*	**No results found**	*-*
**Validation of the MBGR orofacial myofunctional assessment protocol for adults with temporomandibular disorders with disc displacement with reduction**	*Research Article—Two examination forms used in the study: Orofacial Myofunctional Evaluation with Scores (OMES)—MBGR Orofacial Myofunctional Assessment Protocol (MBGR Protocol)*	**MMBRG Protocol—Infants and Preschoolers: Myofunctional Orofacial Clinic Examination**	*Research Article One examination form used in the study: MMBRG Protocol for Infants and Preschoolers*									**Food consumption and masticatory performance of normal weight, overweight and obese children aged 7 to 12 years old**	*Research Article The Expanded orofacial myofunctional assessment protocol (OMES-E) was used*	**Expanded protocol of orofacial myofunctional evaluation with scores: Validity and reliability**	*Research Article The OMES and OMES-E protocols were used*		
**Correlation between scar assessment scales and orofacial myofunctional disorders in patients with head and neck burns**	*Research Article—One examination form used in the study: Orofacial Myofunctional Evaluation with Scores (OMES)*	**Quantitative examination of isometric tongue protrusion forces in children with oro-facial dysfunctions or myofunctional disorders**	*Research Article No examination forms used in the study, only tongue tone was measured*									**Impact of asthma on children´s gustatory sensitivity, masticatory and feeding behaviors**	*Research Article The Expanded orofacial myofunctional assessment protocol (OMES-E) was used*	**Analysis of validity in adults of the expanded protocol of orofacial myofunctional evaluation with scores**	*Conference Abstract Orofacial myofunctional assessment protocol (OMES) was used*		
**Extension and validation of the protocol of orofacial myofunctional assessment for individuals with cleft lip and palate**	*Research Article—One examination form used in the study: MBGR Orofacial Myofunctional Assessment Protocol (MBGR Protocol)*	**Myofunctional orofacial examination: comparative analysis in young adults with and without complaints**	*Research Article One examination form used in the study: Orofacial Myofunctional Evaluation Explanatory Manual*									**Sleep quality and communication aspects in children**	*Research Article ABFW Child Language Test and the MBGR Protocols were used*	**Orofacial myofunctional evaluation with scores in subjects with obstructive sleep apnea**	*Conference Abstract The Expanded orofacial myofunctional assessment protocol (OMES-E) was used*		
**Proposal and content validation of an orofacial myofunctional assessment protocol for individuals with cleft lip and palate**	*Research Article—One examination form used in the study: Orofacial myofunctional assessment protocol for individuals with cleft lip and palate*											**Transcranial direct current stimulation combined with integrative speech therapy in a child with cerebral palsy: A case report**	*Case Report ABFW Child Language Test and OMES Protocol tests were used*	**Electromyographic indices, orofacial myofunctional status and temporomandibular disorders severity: A correlation study**	*Research Article Examination form EMG Test—Research Diagnostic Criteria for TMD (RDC/TMD)—and Orofacial myofunctional assessment protocol (OMES) were used*		
**A one-page orofacial myofunctional assessment form: a proposal**	*Research Article—One examination form used in the study: A one-page orofacial myofunctional assessment form*											**Speech pathology telepractice intervention during the COVID-19 pandemic for Spanish-speaking children with cleft palate: A systematic review**	*Review Article*	**The effect of incentive spirometer training on oromotor and pulmonary functions in children with Down's syndrome**	*Research Article Orofacial myofunctional assessment protocol (OMES) was used*		
**Orofacial myofunctional disorders: guidelines for assessment and treatment**	*Research Article—No examination forms used in the study*													**Orofacial motor functions in pediatric obstructive sleep apnea and implications for myofunctional therapy**	*Research Article EMG Test and Orofacial myofunctional assessment protocol (OMES) were used*		
														**Impaired orofacial motor functions on chronic temporomandibular disorders**	*Research Article EMG Test—Research Diagnostic Criteria for TMD (RDC/TMD)—and Orofacial myofunctional assessment protocols (OMES) were used*		
														**Tongue strength, masticatory and swallowing dysfunction in patients with chronic temporomandibular disorder**	*Research Article Research Diagnostic Criteria for TMD (RDC/TMD)—and Orofacial myofunctional assessment protocol (OMES) and The Iowa Oral Performance Instrument (IOPI) were used*		
														**Chewing in adolescents with overweight and obesity: An exploratory study with behavioral approach**	*Research Article Quality of Mastication Function Questionnaire and The Expanded orofacial myofunctional assessment protocol (OMES-E) were used*		
														**Assessment of the differences in masticatory behavior between male and female adolescents**	*Research Article Quality of Mastication Function Questionnaire and The Expanded orofacial myofunctional assessment protocol (OMES-E) were used*		
														**Muscular and functional changes following adenotonsillectomy in children**	*Research Article Orofacial myofunctional assessment protocol (OMES) was used*		
														**Mastication in overweight and obese children: A comparative cross-sectional study**	*Research Article The Expanded orofacial myofunctional assessment protocol (OMES-E) was used*		
														**The short evaluation of orofacial myofunctional protocol (ShOM) and the sleep clinical record in pediatric obstructive sleep apnea**	*Research Article The Short Orofacial Myofunctional protocol (ShOM) was used*		
														**Ventilatory, phonatory and swallowing impairments in advanced neuromuscular disease patients**	*Research Article Examination form (* +*)*		
														**Orofacial myofunctional therapy in obstructive sleep apnea—Patients’ experiences, adherence to treatment and the importance of trust in the patient—therapist relationship**	*Conference Abstract Expanded orofacial myofunctional evaluation with scores (OMES-E) protocol, the Friedman classification and Tongue range of motion ratio (TRMR) tests were used*		
														**Orofacial rehabilitation after severe orofacial and neck burn: Experience in a Brazilian burn reference centre**	*Research Article The Expanded Protocol of Orofacial Myofunctional Evaluation with Scores (OMES-E) and Mandibular Range of Movement Protocols were used*		
														**An index for the evaluation of 3D masticatory cycles stability**	*Research Article Masticatory Stability Index, (MSI) and Orofacial myofunctional assessment protocols (OMES) were used*		
														**Impact of asthma on children´s gustatory sensitivity, masticatory and feeding behaviors**	*Research Article The Expanded Protocol of Orofacial Myofunctional Evaluation with Scores (OMES-E) was used*		
														**Oral motor and electromyographic characterization of adults with facial fractures: a comparison between different fracture severities**	*Research Article EMG Test and The Expanded Protocol of Orofacial Myofunctional Evaluation with Scores (OMES-E) and Mandibular Range of Movement Protocols were used*		
														**Evaluation of oral-motor movements and facial mimic in patients with head and neck burns by a public service in Brazil**	*Research Article Clinical Score for Facial Mimic Protocol and The Expanded Protocol of Orofacial Myofunctional Evaluation with Scores (OMES-E) and Mandibular Range of Movement Protocols were used*		
														**Predictors of uvulopalatopharyngoplasty success in the treatment of obstructive sleep apnea syndrome**	*Research Article Polysomnographic evaluation, OMES and a dynamometer (force transducer for maximal isometric tongue strength) protocols were used*		
														**Masticatory muscle function three years after surgical correction of class III dentofacial deformity**	*Research Article EMG test was used*		
														**Sleep quality and communication aspects in children**	*Research Article ABFW Child Language Test and the MBGR Protocols were used*		
														**Congenital heart disease in children: Orofacial myofunctional aspects, eating behavior and facial temperature**	*Research Article A questionnaire to assess eating behaviors (Montreal Children's Hospital Feeding Scale) and Expanded orofacial myofunctional assessment protocol (OMES-E) and Child Language Test (ABFW), and Thermography infrared of facial temperature protocols were used*		
														**Changes in jaw and neck muscle coactivation and coordination in patients with chronic painful TMD disk displacement with reduction during chewing**	*Research Article EMG Test—Diagnostic Criteria for TMD (DC/TMD)—and Orofacial myofunctional assessment protocols (OMES) were used*		
														**Holistic semi-presential evaluation of oropharygeal dysphagia with the framework of International Classification of Functioning, Disability and Health: Optimizing evaluation to improve rehabilitation treatment**	*Research Article The International Classification of Functioning, Disability and Health (ICF) protocol was used*		
														**Normalizing surface electromyographic measures of the masticatory muscles: Comparison of two different methods for clinical purpose**	*Research Article EMG test and DC/TMD protocols were used*		
														**Effect of treatment of dentofacial deformity on masseter muscle thickness**	*Research Article Ultrasonographic evaluation was used*		
														**Speech pathology telepractice intervention during the COVID-19 pandemic for Spanish-speaking children with cleft palate: A systematic review**	*Review Article*		
														**Acoustic vocal measures in women without voice complaints and with normal larynxes**	*Research Article Auditory screening ‘consisted of air conduction pure tone scans at 500, 1000, 2000 and 4000 Hz (25 db)’ was used*		
														**Surface electromyography and magnetic resonance imaging of the masticatory muscles in patients with arthrogenous temporomandibular disorders**	*Research Article RDC/TMD protocol and EMG test and MRI were used*		
														**Surface electromyographic assessment of patients with long lasting temporomandibular joint disorder pain**	*Research Article RDC/TMD protocol and EMG tests were used*		
														**Mandibular kinematics and masticatory muscles EMG in patients with short lasting TMD of mild-moderate severity**	*Research Article RDC/TMD protocol and EMG tests were used*		
														**Dynamic Evaluation of Motor Speech Skill: Adaptation for Brazilian Portuguese**	*Research Article Dynamic Evaluation of Motor Speech Skills—DEMSS was used*		
														**Reorganization of muscle activity in patients with chronic temporomandibular disorders**	*Research Article RDC/TMD protocol and EMG tests were used*		
														**Nasal patency and otorhinolaryngologic-orofacial features in children**	*Research Article MBGR protocol Peak Nasal Inspiratory Flow (PNIF) were used*		

## Results

Among all studies, OMES and OMES-E tests were the most preferred myofunctional anamnesis protocols. MGBR was following them. A few studies were based on conventional conventional OMD examination rules. It was observed that some studies examining myofunctional anomalies and temporomandibular disorders together used DC/TMD or RDC/TMD and, EMG tests. Some studies were specifically aimed at measuring the functions and working force of the tongue. Two researchers have published publications promoting their personal measurement protocols. One study used a protocol to evaluate facial muscular mimic-specific measurements. A nutrition scale was used in one study. Nasal obstruction was separately evaluated in one study.

In some studies, specialized tests were used to evaluate specific functions, such as the ability to produce speech sounds. It was observed that studies examining OMD and TMD together also used RDC/TMD and EMG tests. Some studies were specifically aimed at measuring the functions and functional strength of the tongue. Some researchers have published publications promoting their own personal measurement protocols. One study used a protocol that made mimic-specific measurements. A nutrition scale was used with MOE in one study. Effects of nasal obstruction was evaluated by using a MOE in one study.

The MOE examination forms used in the screened studies included observation of the patient's orofacial posture at rest, assessment of swallowing patterns, assessment of speech production, and a comprehensive oral-motor examination. Those included assessments of various aspects of orofacial function, such as lip and tongue resting posture, swallowing patterns, and speech production. In OMES protocol each assessment is given a score to define the severity of orofacial dysfunctions which are observed and detected. It is observed that orofacial myofunctional examinations for the studies screened were performed in a clinical setting such as language-speech pathology clinic or dental clinic or ENT clinics or pediatric clinics, which shows that the subject is definitely multi and interdisciplinary.

## Discussion

This literature review has shown us that the MOE protocol is used in many different disciplines of medicine and dentistry and in the diagnosis and treatment of many different orofacial anomalies. The aims and results of all the publications scanned in the study are given below in the form of a short review. In addition, which MOE test was used in each study is indicated in Table 1 one by one.

De Araújo SRS et al. [28] investigated orofacial myofucntional structures of old people with Parkinson’s disease and found that orofacial functions worsen in elder people with Parkinson’s disease.

Bueno et al. [29] conducted a study to validate MGBR protocol for adults patients with TMD and proved the validation of use TMJD and OMD patients.

In their study, Magnani et al. [30] used OMES and two different scar scales in patients with head and neck burns to evaluate the OMD. They presented a correlation between scar severity in patients with burn and OMD presence.

Graziani et al. [31, 32] studied the validation of MBGR protocol in cleft lip and palate patients. They presented that the protocol was valid entirely in let lip and palate patients.

Paskay LC [33]. in her study, presented a one page orofacial myofunctional examination form of hers own.

In his study, Hanson ML [34]. drew a general framework for the content rather than giving a form suggestion. The author mentioned that, patients should be seen for repeated examination for at least two years or until all orthodontic work has been completed. Treatment of orofacial myofunctional disorders gives the most successful results when the clinician, child, parent, patient, and dentist work in close collaboration.

Medeiros et al. [35] studied the validity of MBRG in infants and preschoolers and found the results as good and even excellent in agreement. An erratum was published for the study one year later.

Rohrbach et al. [36] examined the isometric tongue protrusion forces (TPF) of children with OFD and controls. They found that subjects with OFD show significantly lower proficiency in the accuracy and durability of tongue projection forces. This may have a high impact on the phenotyping of children with OFD and influence therapeutic approaches.

Macedo et al. [37] conducted a study to confirm myofunctional orofacial features in young adults and to compare data from individuals with and without myofunctional complaints. They reported that major myofunctional orofacial changes in young adults with complaints may be related to changes in mandibular movements and chewing or swallowing patterns that reflect key elements of clinical assessment. Many examination and characterization parameters do not change between groups and should be analyzed for relevance.

Lima et al. [38] evaluated the effect of transcranial direct current stimulation and integrative talk therapy combination in a child with cerebral palsy. The combination therapy process contributed to improving rehabilitation of speech outcomes in a cerebral palsy patient.

Pimenta et al. [39] presented an index (Masticatory Stability Index, MSI) which aims to analyze the stability of chewing cycles in same conditions and to examine it in a patient group of subclinical mild temporo-mandibular disorder (TMD). They stated that MSI was effective for measuring the stability of chewing cycles.

Barbosa et al. [40] investigated the relation between congenital heart disease and eating behavior and found that eating difficulties were more frequent amongst those. Furthermore, the authors reported that there were changes in orofacial myofucntional structure and behaviors and, posture of those when compared to the control groups.

Santos et al. [41] studied food intake and chewing activity in normal weight, overweight and obese children aged 7–12 years and they concluded that there were differences between obese and normal weight children regarding food consumption and masticatory activity.

In their study, Arias Gullien et al. [42] assessed the effect of astma on taste sensitivity, chewing behavior and eating problems among children. The authors mentioned that asthmatic children showed differences in taste sensitivity, and masticatory and feeding behaviors.

De Castro Correa et al. [43] investigated the relation between quality of sleep and communication skills inn children and found a correlation between sleep quality and communication skills related to myofunctional orofacial aspects.

In their case report, Carvalho Lima et al. [44] studied the results of integrative speech therapy combination with ‘anodal transcranial direct current stimulation’ and reported promising findings.

In their systematic review study, Palomares-Aguilera M et al. [45] investigated the effects of telepractice for speech pathologies for Spanish speaking children with cleft palate during Covid-19 pandemic. They concluded that audiovisual materials are very useful for telepractice in the rehabilitation of such individuals.

In their study, in which they compared orofacial myofunctional evaluation with scores protocol with traditional orofacial myofunctional evaluation, Felicio and Ferreira [42] proved the validity and reliability of OMES protocol.

In an other study, de Felicio CM et al. [18, 46] expanded OMES as OMES-E and investigated the validity and reliability of OMES-E. They mentioned that, regarding the clinical necessity of a more detailed and precise assessment of OMD for diagnosis and monitoring of responses to therapy, they modified OMES protocol and expanded OMES protocol as OMES-E in terms of the number of items to be evaluated and the width of its numerical scales. So that, diagnostic accuracy could be increased with more expanded numerical evaluation scales. The authors confirmed the reliability and validity of the OMES-E protocol.

Folha et al. [47] in their study, aimed to analyze the validity and the reliability of the OMES-E protocol in adults. They reported that the protocol was vail and reliable with good sensitivity, specificity, accuracy and predictive values.

Folha et al. [48] in their other study, aimed to examine if OMES-E protocol can differentiate the myofunctional orofacial features between healthy people and patients with OSA or not. They resulted that OMES-E protocol has ability for such a discrimination.

De Felicio CM et al. [49] investigated the correlation between surface electromyography (EMG) of chewing muscles, orofacial myofunctional status and temporomandibular joint disorder (TMD) severity scores. They reached significant correlations between these protocols.

Ibrahim AF et al. [50] examined the effect of incentive spirometry training on orofacial myofunctions and pulmonary functions in children with Down's syndrome. Orofacial myofunctional training exercises are more effective than stimulating spirometry training to improve both pulmonary and oromotor functions in children with Down's syndrome.

De Felicio et al. [51] conducted a study to identify correlation between orofacial myofunctional scores and sleeping disorders: obstructive sleep apnea and primary snoring. They concluded that children with tonsillar hypertrophy and OSA had related impairments in orofacial myofunctional functions and less muscular coordination than children with primary snoring.

Ferreira et al. [52] assessed the myofunctional orofacial functions of patients with moderate to severe signs and symptoms of chronic TMJD by using surface electromyography (EMG) of dynamic rhythmic muscular activities. They found that the alterations of orofacial motor control in patients with moderate to severe chronic TMJD was characterized by impaired orofacial functions and increased activity of the muscles of balancing sides during unilateral chewing.

Marim et al. [53] in their study, aimed to examine whether tongue strength, masticatory difficulties, and orofacial myofunctional were associated. They concluded that, patients with chronic TMD had lower tongue strength and worse chewing and swallowing functions, and these parameters were interrelated.

Pedroni-Pereira A et al. [54] evaluated objective, subjective and behavioral aspects of masticatory function in overweight and obese adolescents. They found that adolescents with high-weight presented changes in chewing behavior and more difficulty during the masticatory function compared to normal-weight ones.

Scudine KGO et al. [55] examined the effects sexual difference on chewing performance of adolescents. They reported alterations in myofunctional aspects between male and female objects.

Bueno Dde A et al. [56] evaluated myofunctional orofacial changes in children before and after adenotonsillectomy. The authors said that they had seen partial recovery and positive changes in myofunctional orofacial behaviors and functions, especially in the mobility and posture, of objects.

Santos REA et al. [57] analyzed the chewing patterns in normal-weight, over-weight and obese children and they stated that obese children had masticatory changes such as: big-sized bites, lesser chewing time and quick eating which could contribute to obesity.

Correa CC et al. [58] examined the relation between OSA and myofucntional orofacial status of children. The authors found changes in rest and deglutition position of tongue. The authors offered the sleep clinical records to be evaluated with the findings of myofunctional orofacial examination findings in the evaluation of OSA in children.

Muñoz-Vigueras N et al. [59] examined respiratory, phonatory and swallowing disorders and their relationship to physical condition in a group of advanced Neuromuscular Diseases (NMD). Patients in lower physical status are at higher risk of suffering from ventilation, phonatory and deglutition complications after NMD is diagnosed.

Hansen D et al. [60] mentioned that, orofacial myofunctional therapy (OMT) is promising in treatment strategy of OSA based focused exercises. Furthermore, trust in the patient-therapist relationship is a major factor for sucsessful and adequqte results. The authors aimed to evaluate experiences and adherence to OMT and the effects of a trust relationship between the therapists and motivated OSA patients.

Magnani DM et al. [61] aimed to assess the advantages of a myofunctional orofacial rehabilitation protocol for pathologies caused by full thickness orofacial and neck burns, comparing the results regarding early and late intervention. The findings of their study indicated that non-invasive orofacial myofunctional management of contractures is effective for patients with orofacial and neck burns including those which had long term sequelae.

Da silva AP et al. [62] compared the oral motor performance and function between two different facial fracture groups. They presented that severity of facial fractures did not have big impact on functioning and performance orofacial myofunctions 4 months after the correction of fractures.

Magnani DM et al. [63] assessed the features of oral-motor movements and facial mimic of patients who had head and neck burns. The authors stated that, patients with head and neck burns had significant deficiencies related to posture, position and mobility of the orofacial myofunctional tissues, including facial movements.

Braga et al. [64] aimed to examine the correlation between uvulopalatopharyngoplasty success and caraniofacial hard tissue posture and orofacial myofunctional status in patients with OSA. The authors only determined an increase in the strength of anterior portion of the tongue of patients with surgical success.

Trawitzki LV et al. [65] evaluated the effect of interdisciplinary treatment approach in patients with dentofacial deformities with regard to electromyographic activity (EMG) of chewing muscles three years after surgical correction. The authors reported an increase in the EMG activity of the chewing muscles, mainly in the masseter muscle, with similar values compared to the controls’.

Fasscollo CE et al. [66] using electromyographic (EMG) analysis, investigated the behavior of the anterior temporalis, masseter, and sternocleidomastoid muscles during chewing in time and frequency domains in patients with chronic painful Temporomandibular disorder with reduced disc displacement (TMD-DDR). They reported that patients with TMD-DDR with chronic pain during chewing showed changes in the jaw and neck muscles with greater impairment of the old function specific to chewing.

Renom-Guiteras M Et al. [67] aimed to present the design of a model for the holistic evaluation of oropharyngeal dysphagia that takes into account the components of the 'International Classification of Functioning, Disability and Health (ICF)' and that can be applied both face to face and remotely using Information and Communication Technology (ICT) tools. They stated that examination of oropharyngeal dysphagia should be within the biopsychosocial framework pointed by the ICF.

Mapelli A et al. [68] aimed the comparison of two different methods, a new normalization method (wax pad, WAX) with the currently used cotton roll (COT) technique, in surface electromyography of the masticatory muscles. The authors reported that the WAX normalization method is as stable as the COT method but more reproducible and more convenient.

Trawitzki LV et al. [69] aimed to determine the changes in thickness of masseter muscle before and after interdisciplinary orthodontic, orofacial myofunctional and surgical treatment of class III dentofacial skeletal anomalies. The authors concluded that, an increase was observed in masseter thickness after the surgical correction of class III accompanied by interdisciplinary protocols.

Finger LS et al. [70] aimed to describe the voice measures of young women who has normal larynxes without any voice problems. As the result they found all the values within and out of normal distribution showed similar results to the samples presented in the national and international literature.

Lodetti et al. [71] aimed to confirm the features of surface electromyography (sEMG) of masticatory muscles in patients with temporomandibular disorders (TMDs) with several pathology. The authors reported recording of the masticatory muscle function through sEMG as to be first diagnostic method for patients with TMDs, in accorddance with MRI assessment in selected cases.

Tartaglia GM et al. [72] compared normalized electromyographic (EMG) characteristics of chewing muscles in patients with temporomandibular joint disorders (TMD) with healthy controls’. They found that young adult patients with chronic TMJD have an increased and more asymmetric standardized activity in their anterior temporal muscle, and lesser mean power frequencies, when compared to healthy controls.

De Felicio CM et al. [73] examined mandibular functional and standardized surface electromyography (sEMG) features of chewing muscles of subjects with short period TMJD of both mild and moderate severity. They presented that, in general, TMJ subjects has similarities with the controls in several functional parameters, and the EMG results of the static test, but there were some changes in the chewing.

Gubani MB et al. [74–76] in their study, adapted Dynamic Evaluation of Motor Speech Skills—DEMSS (Strand et al., 2013; Strand & McCauley, 2019) test for Brazilian Portuguese (BP). The authors validated the translation and adaptation of the test.

Mapelli A et al. [77] aimed to examine reorganization of muscle activity in patients with long lasting temporomandibular joint disorders (TMJD) and, if there is, how it is affected by severity of symptomatology. The authors presented that, chronic TMJD patients, especially those with severe symptoms had a reorganized activity, which resulted in worse kinematic performances.

Milanesi JM et al. [78] compared nasal passage patency and otolaryngologic-orofacial features in children. Lower nasal patency was observed in children with restless sleep, accompanied by signs and symptoms of rhinitis, decrease in hard palate, changes in mastication, deglutition and speech functions. It is also underlined that most of the children had allergic rhinitis signs and symptoms.

Another problem that can be caused by myofunctional orofacial anomalies is orofacial pain. In the modern world, myofascial pain is one of the most common problems related to the orofacial region, both due to our head and body postural disorders, due to stress and myofunctional orofacial anomalies. Orzeszek et al. [79] In their literature study, drew attention to orofacial myalgia and myofascial pain and examined the methods used for treatment in this regard.

Since this study was conducted to determine which OMES test is the most known and preferred in the literature, the quality of the methodologies of the studies was not examined one by one. This was a limitation of the study. Studies in the field about orofacial disorders, which is a highly multidisciplinary medical problem, are relatively insufficient. Myofunctional orofacial dysfunction is a complex condition that requires careful examination and appropriate multidiscipliner treatment to achieve optimal outcomes. Anomalies that occur in a region that we call the stomatognathic system and where vital and important functions such as speech, swallowing, feeding, breathing, chewing, facial appearance and posture are performed, cause many other medical problems and even anomalies of other systems. Furthermore studies with future studies could develop the evaluation and rehabilitation processes.

## Conclusion

The MOE is a valuable tool in many disciplines of dentistry and medicine to assess the orofacial region and to identify orofacial myofunctional disorders both statically and dynamically. In general, across all disciplines, OMES was found to be the most preferred test. In addition, it has taken its place in the literature that the OMES test makes consistent and reliable measurements in many different conditions.

## Data Availability

The datasets used and/or analyzed during the current study are available from the corresponding author on reasonable request.
